# Circ_0000396 inhibits rheumatoid arthritis synovial fibroblast growth and inflammatory response via miR-203/HBP1 axis

**DOI:** 10.1186/s40709-020-00131-4

**Published:** 2021-01-06

**Authors:** Laifang Wang, Qing Zhao, Na Wang, Yanjie Ding, Lingli Kong, Jing Wang

**Affiliations:** Department of Rheumatism and Immunology, Huaihe Hospital of Henan University, No. 115 Ximen Street, Kaifeng, 475000 Henan China

**Keywords:** circ_0000396, Rheumatoid arthritis, Synovial fibroblasts, miR-203; HBP1

## Abstract

**Background:**

Circ_0000396 was found to be down-regulated in the rheumatoid arthritis (RA) patients and had a high diagnostic value. However, the function and mechanisms underlying circ_0000396 in RA progression remain unclear.

**Methods:**

The expression of circ_0000396, microRNA (miR)-203 and HMG-box transcription factor 1 (HBP1) was detected using qRT-PCR and western blot. The proliferative and apoptotic capabilities of rheumatoid arthritis synovial fibroblasts (RASFs) were measured by colony formation, CCK-8, flow cytometry and western blot assays, respectively. The levels of interleukins (IL)-6, IL-1β, IL-8 and tumor necrosis factor-α (TNF-α) were detected using enzyme-linked immunosorbent assay (ELISA). The target correlations between miR-203 and circ_0000396 or HBP1 were validated using pull-down and dual-luciferase reporter assay.

**Results:**

Circ_0000396 was decreased in RA synovial tissues and RASFs, and overexpression of circ_0000396 suppressed cell proliferation, induced cell apoptosis and reduced the release of inflammatory cytokine IL-6, IL-1β, IL-8 and TNF-α in RASFs, while circ_0000396 deletion functioned oppositely. MiR-203 was confirmed to be a target of circ_0000396, and miR-203 reversed the protective effects of circ_0000396 on the dysfunction and inflammation of RASFs. HBP1 was a target of miR-203, and silencing miR-203 inhibited RASFs malignant changes by regulating HBP1. In addition, circ_0000396 could regulate HBP1 by sponging miR-203, and HBP1 decrease attenuated the effects of circ_0000396 on RASF growth and inflammation.

**Conclusion:**

Circ_0000396 inhibited the growth and inflammation in RASFs by regulating miR-203/HBP1 axis, providing a potential therapeutic target for RA.

## Background

Rheumatoid arthritis (RA) is a chronic, autoimmune, systemic disorder that is characterized by hypertrophy, hyperplasia, and angiogenesis of synovial tissues [1, 2]. The pathogenesis of RA is complex; increasing evidence suggests that synovial fibroblasts of RA (RASFs), also known as fibroblast-like synoviocytes, perform important effects on the pathogenesis of RA [3, 4]. It is reported that RASFs have similar characteristics to tumor cells, such as tumor-like proliferation, migration, invasion, and resistance to apoptosis [5, 6]. Besides, over-secretion of cytokines in RA is another key feature of RASFs, which activates the immune responses, and in turn, stimulate RASFs, ultimately leading to joint destruction [7]. Thus, a better knowledge of RASFs is necessary for improving the therapy of RA.

Circular RNAs (circRNAs) are a category of endogenous RNAs with conserved closed continuous loops structures, and widely exist in the eukaryotic transcriptome [8]. Recent studies have found that circRNAs are the potential regulators in a variety of diseases, including cancers, cardiovascular and autoimmune diseases, by regulating numerous biological events in cells [9–11]. Additionally, the involvement of circRNAs in the progression of RA also was identified [12]. For example, Luo et al*.* [13] proved that hsa_circ_0044235 in peripheral blood was the potential biomarker for the diagnosis of RA patients. Li et al*.* [14] found circFADS2 knockdown protected chondrocytes against extracellular matrix (ECM) degradation, inflammatory cytokines release, and apoptosis by modulating miR-498/mTOR pathway. Li et al*.* [15] demonstrated hsa_circ_0001859 up-regulated ATF2 through miR-204/211 to stimulate inflammation in SW982 cells. Thus, circRNAs may be promising candidates for the treatment of RA. Circ_0000396 is a novel identified circRNA; it has been found that it was downregulated in the RA patients versus the healthy group, and showed a higher receiver operating characteristic (ROC) area under the curve (AUC), while circ_0000396 might be a potential diagnostic biomarker for RA patients [16]. However, the roles of circ_0000396 in RASF dysfunction remain unclear.

This study mainly focused on identifying the expression patterns of circ_0000396 in RA synovial tissues and RASFs, exploring the roles and the underlying mechanism of circ_0000396 in RASF biological behavior and inflammation.

## Methods

### Clinical samples

Samples of RA synovial tissues were collected from 31 RA patients who received knee joint replacement surgery at Huaihe Hospital of Henan University. All RA patients were diagnosed in line with the American College of Rheumatology classification. For non-RA control, the synovial tissue samples were collected from 25 patients who underwent emergency trauma amputation. None of the healthy controls had RA or osteoarthritis. All samples were stored in liquid nitrogen for further analysis.

### Cell culture

RA samples and healthy control samples were cut into small pieces. After washing with Hank’s solution, all pieces were digested with trypsin solution. Subsequently, RASFs and normal synovial cells (NSFs) were separated using the fluorescence activated cell sorting (FACS) technique based on the expression of CD68 (a macrophage marker) and CD90 (a fibroblast marker) (Additional file 1: Fig. S1). All collected cells were grown in DMEM (Gibco, Carlsbad, CA, USA) harboring 10% FBS and incubated with 5% CO_2_ at 37 °C.

### Cell transfection

The mimic or inhibitor targeting miR-203 (miR-203 or in-miR-203), and the corresponding negative control (miR-NC and in-miR-NC) were purchased from RIBOBIO (Guangzhou, China). The small interfering RNA (siRNA) targeting circ_0000396 covalent closed junction (si-circ_0000396), siRNA targeting HMG-box transcription factor 1 (HBP1) (si-HBP1), siRNA negative control (si-NC), empty vector (pcDNA), pcDNA-circ_0000396 overexpression vector (circ_0000396), pcDNA-HBP1 overexpression vector (HBP1) were designed by Genepharma (Shanghai, China). Cell transfection was carried out with Lipofectamine 3000 (Invitrogen, Carlsbad, CA, USA).

### RNA isolation and qRT-PCR

Total RNA was prepared by using TRIzol reagent (Invitrogen, Carlsbad, CA, USA) following the standard procedure. Then extracts were interacted with Rnase R (Epicentre, Madison, WI, USA), and the yield and purity were measured by a NanoDrop 2000 instrument (Thermo Fisher Scientific, Rochester, NY, USA). Subsequently, first strand cDNA was generated using a PrimeScript RT Reagent Kit (Takara, Dalian, China), and then qRT-PCR was conducted with SYBR Green methods (Takara, Dalian, China) on the ABI7500 system (Applied Biosystems, Foster City, CA, USA). The relative fold changes in expression were assessed using 2^−ΔΔCt^ method and normalized by GADPH and U6. The experiment was performed in triplicate, and the average was taken. The primers were presented as follows: circ_0000396: F 5΄- GCTCACACCAATCCCCTG-3΄, R 5΄-GCCTGGCACACAGTAGACAC-3΄; GADPH: F 5΄-GATATTGTTGCCATCAATGAC-3΄, R 5΄-TTGATTTTGGAGGGATCTCG-3΄; miR-203 F 5΄-TGCTCTAGAGGCGTCTAAGGCGTCCG-3΄, R 5΄- CCCAAGCTTCACCTCCCAGCAGCACTTG-3΄; HBP1: F 5΄- TGAAGGCTGTGATAATGAGGAAGAT-3΄, R 5΄- CATAGAAAGGGTGGTCCAGCTTA-3΄; U6: F 5΄-CTCGCTTCGGCAGCACA-3΄, R 5΄-ACGCTTCACGAATTTGCGT-3΄.

### Cell proliferation analysis

For colony formation assay, transfected RASFs were seeded into a 12-well plate at a density of 1000 cells per well and then cultured in a CO_2_ incubator at 37 °C for 2 weeks. Subsequently, cells were washed with phosphate-buffered saline (PBS), and then fixed with 4% paraformaldehyde for 15 min and stained with 0.5% crystal violet for 20 min. The colonies were photographed and imaged using a microscope reader (Bio-Rad, Hercules, CA, USA). The experiments were replicated at least three time.

For CCK-8 assay, RASFs were seeded into 96-well plates with 5000 cells per well overnight following transfection, and then incubated with 10 μL CCK-8 solution (Dojindo Laboratories, Kumamoto, Japan) for 2 h. The absorbance of each well at 450 nm was determined by a microplate reader in the indicated time. Each sample was prepared in quadruplicate, and the entire experiment was repeated three times.

### Western blot

Total proteins were prepared using RIPA buffer, and then qualified by the bicinchoninic acid (BCA) method. Subsequently, the extracted proteins were separated by 10% SDS-PAGE, and afterwards shifted to a polyvinylidene difluoride membrane (Millipore, Billerica, MA, USA). Then immunoblotting was performed with the antibody against HBP1 (1:5000, ab83402, Abcam, Cambridge, MA, USA), Ki67 (1:5000, ab16667, Abcam), cleaved PARP (1:3000, ab32064, Abcam), cleaved caspase3 (1:3000, ab2302, Abcam), and β-actin (1:1000, Cat#4967, Cell Signaling Technology, Boston, MA, USA), followed by interaction with HRP-conjugated secondary antibody and visualized by electrochemiluminescence. Each sample was prepared in triplicate, and the same experiment was repeated three times.

### Cell apoptosis assay

After transfection, a total of 1 × 10^5^ RASFs from each group were washed with PBS. Then, cells were collected and resuspended in binding buffer, followed by incubation with annexin V-FITC (10 μL) and PI (10 μL) in the dark (BD Biosciences, San Jose, CA, USA). Finally, apoptotic cells were analyzed by a flow cytometer with Flow J software (FACSCanto™ II, BD Biosciences, San Jose, CA, USA). In each independent experiment, three parallel wells were made, and the procedures were carried out in triplicate.

### Enzyme linked immunosorbent assay (ELISA)

Cytokine concentrations from the harvested supernatants of RASFs after appropriate treatment were measured by using commercial interleukins IL-6, IL-1β and IL-8 and tumor necrosis factor-α (TNF-α) ELISA kits (R&D Systems, Minneapolis, USA) according to the instructions of the manufacturer. Each sample was prepared in triplicate, and the experiment was repeated three times.

### Dual-luciferase reporter assay

The circinteractome (https://circinteractome.nia.nih.gov/) and DIANA TOOLS databases (http://diana.imis.athena-innovation.gr/DianaTools/index.php) were used to predict potential binding sites. The wild-type (WT) or mutant (MUT) circ_0000396 and HBP1 3΄ UTR containing the potential binding sites of miR-203 were cloned into the pmiR-RB-Report luciferase vector (Promega, Shanghai, China) to generate the wild-type pmiR-RB-Report-circ_0000396/HBP1 3΄ UTR plasmids (circ_0000396 WT or HBP1 3΄ UTR WT) or mutated type pmiR-RB-Report-circ_0000396/HBP1 3΄ UTR plasmids (circ_0000396 MUT or HBP1 3΄ UTR MUT), respectively. Subsequently, RASFs were co-transfected with these constructed luciferase plasmids and miR-203 mimic or miR-NC using Lipofectamine™ 3000 (Invitrogen, Carlsbad, CA, USA) for 48 h. Finally, the luciferase activity was detected by a dual luciferase assay kit (Promega, Shanghai, China) and normalized with Ranilla luciferase activity.

### Pull-down assay

Biotin-labeled miR-203 (Bio-miR-203), Bio-miR-203 MUT, and Bio-miR-NC generated by GenePharma were transfected into RASFs for 48 h. Then RASFs were lysed, and cell lysates were incubated with Dynabead M-280 streptavidin beads (Invitrogen, Carlsbad, CA, USA) overnight. After elution, the bead-bound was purified using TRIzol and analyzed using qRT-PCR.

### Statistical analysis

Data was manifested as mean ± standard deviation (SD) and analyzed using GraphPad Prism 7 software (GraphPad, San Diego, CA, USA). The differences in different groups were analyzed by Student’s *t* test, Mann–Whitney U-test or one-way analysis of variance (ANOVA) according to the data normality. *p* value less than 0.05 exhibited a statistical significance.

## Results

### The expression of circ_0000396 in RA synovial tissues and RASFs

**Fig. 1:**
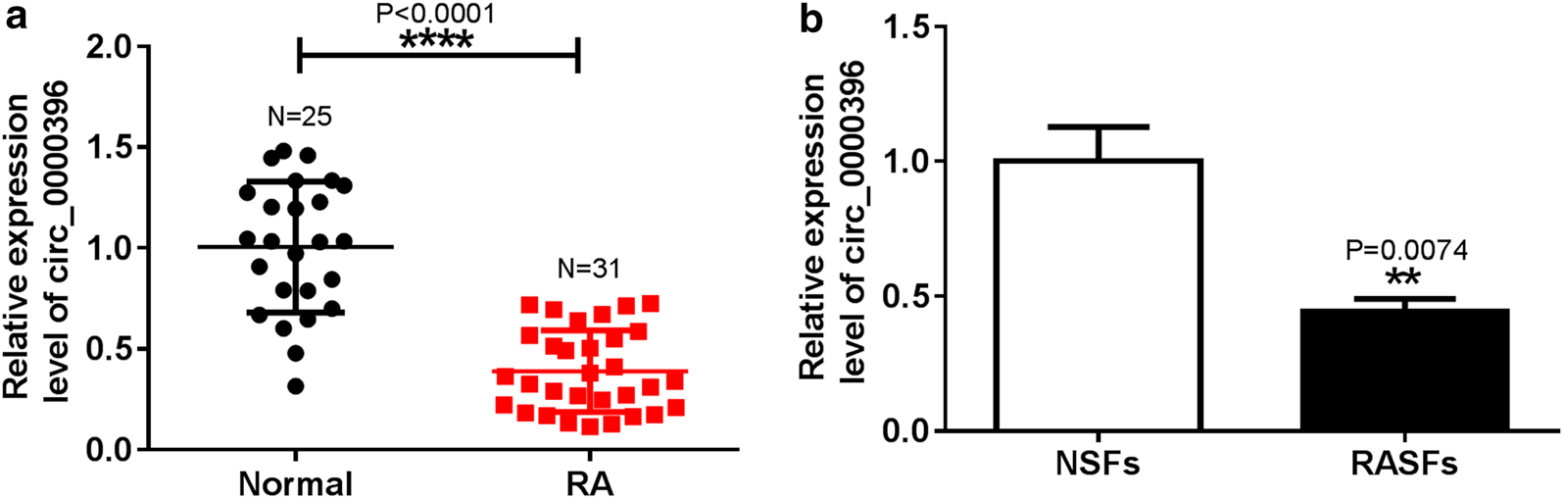
The expression of circ_0000396 in RA synovial tissues and RASFs. **a** Expression of circ_0000396 in RA synovial tissues and healthy control tissues was measured by qRT-PCR. **b** qRT-PCR analysis of circ_0000396 expression in RASFs and NSFs was performed. ^*^p < 0.05, ^**^p < 0.01, ^***^p < 0.001, ^****^p < 0.0001 (Student’s *t* test)

To investigate the expression profile of circ_0000396 in RA, qRT-PCR analysis was performed. The results showed that circ_0000396 expression was significantly decreased in synovial tissues with RA relative to these in non-RA controls (Fig. 1a). Besides, it was also found that circ_0000396 expression was lower in RASFs compared with that in NSFs (Fig. 1b). These results indicated that circ_0000396 expression might be associated with the progression of RA.

### Effects of circ_0000396 on the growth and inflammation of RASFs

**Fig. 2:**
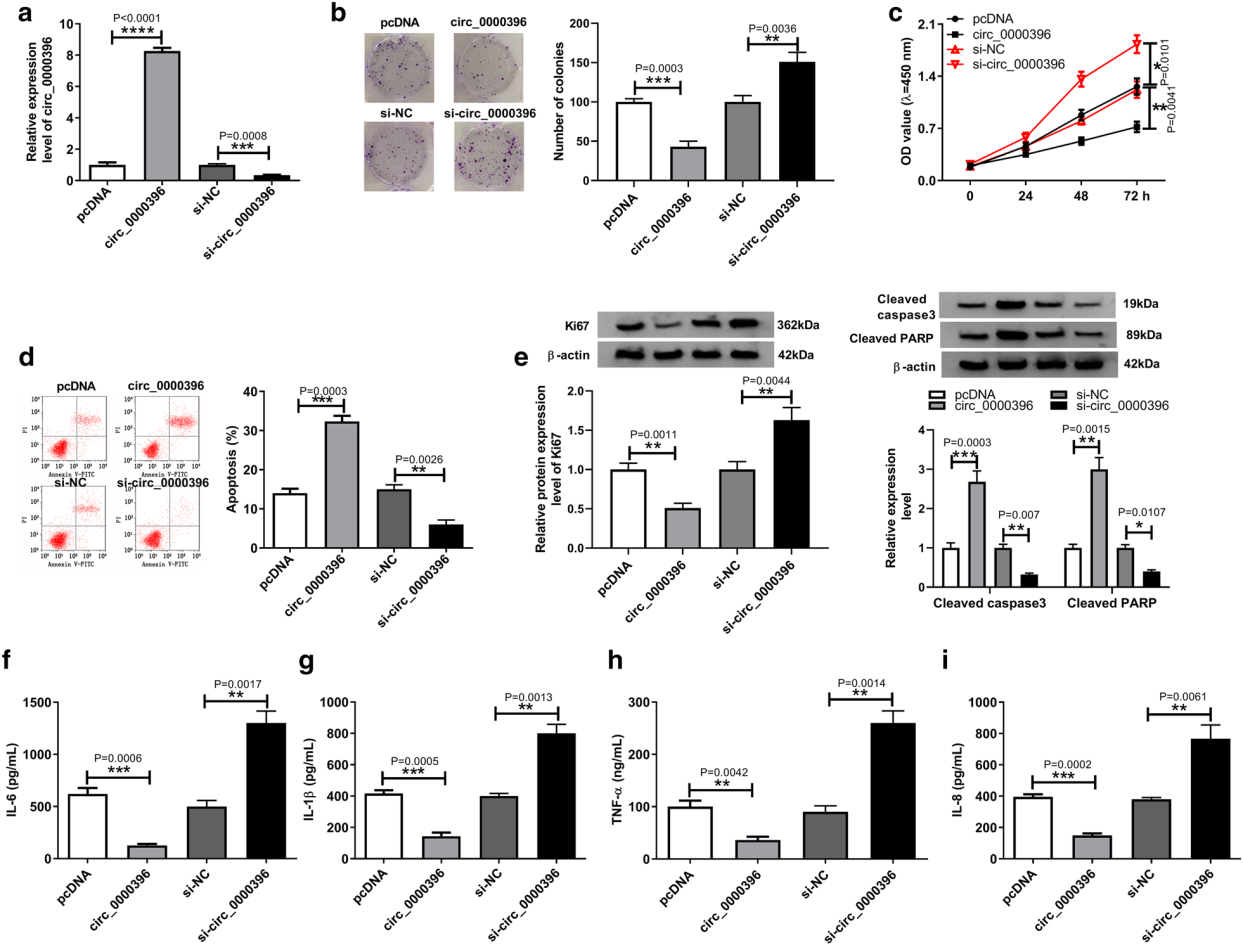
Effects of circ_0000396 on the growth and inflammation of RASFs. RASFs were transfected with pcNDA, circ_0000396, si-NC, or si-circ_0000396. After transfection, **a** circ_0000396 expression was detected by qRT-PCR (Student’s *t* test); **b**, **c** the proliferation of RASFs was analyzed using colony formation assay and CCK-8 assay, respectively (Mann–Whitney U-test); **d** the number of apoptotic RASFs was examined by flow cytometry (Student’s *t* test); **e** western bolt analysis of Ki67, cleaved caspase3 and cleaved PARP expression in RASFs was performed (Student’s *t* test); **f**–**I** the level of inflammatory cytokines IL-6, IL-1β, IL-8 and TNF-α in RASFs was measured using ELISA assay (Student’s *t* test). ^*^p < 0.05, ^**^p < 0.01, ^***^p < 0.001, ^****^p < 0.0001

To identify the potential role of circ_0000396 in the progression of RA, circ_0000396 was up-regulated or down-regulated in RASFs by transfecting with circ_0000396 or si-circ_0000396. As expected, circ_0000396 pcDNA notable increased the expression of circ_0000396, while si-circ_0000396 transfection effectively decreased its expression in RASFs, and transfection of negative control had no effect on circ_0000396 expression (Fig. 2a). After that, results of colony formation and CCK-8 assay showed circ_0000396 up-regulation significantly inhibited colonies formation and the proliferation of RASFs, while circ_0000396 down-regulation promoted colonies formation and RASF proliferation (Fig. 2b, c). Inversely, the number of apoptotic RASFs was increased by circ_0000396 overexpression and was decreased by circ_0000396 knockdown (Fig. 2d). Moreover, western blot analysis displayed that circ_0000396 up-regulation decreased Ki67 level and increased cleaved PARP and cleaved caspase3 expression levels in RASFs, while circ_0000396 down-regulation showed inverse effects (Fig. 2e), further indicating circ_0000396 suppressed cell proliferation and induced apoptosis in RASFs. Meanwhile, the expression of inflammatory cytokines IL-6, IL-1β, IL-8 and TNF-α was detected using ELISA, and results displayed that, by contrast with the negative control, the secretion levels of IL-6, IL-1β, IL-8, and TNF-α were suppressed by overexpressed circ_0000396, but was elevated by the depletion of circ_0000396 (Fig. 2f–i). Taken together, circ_0000396 inhibited cell proliferation and inflammatory response, but induced cell apoptosis in RASFs.

### MiR-203 is identified as a direct target of circ_0000396

**Fig. 3:**
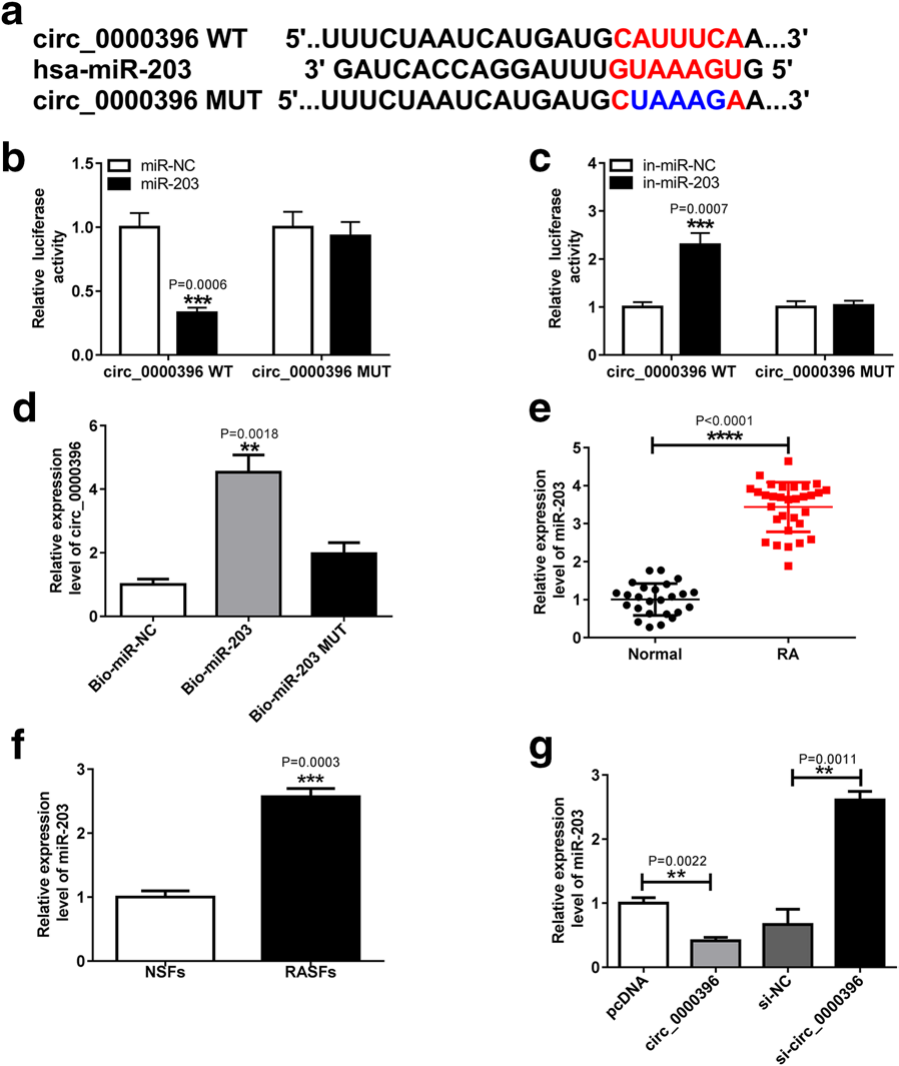
MiR-203 is identified as a direct target of circ_0000396. **a** The putative binding sits between circ_0000396 and miR-203 were predicted. **b**–**d** The interaction between circ_0000396 and miR-203 was confirmed by the dual-luciferase reporter assay (Mann–Whitney U-test) and pull-down assay in RASFs (ANOVA). **e**, **f** Expression of miR-203 in RA synovial tissues and healthy control tissues as well as in RASFs and NSFs was detected by qRT-PCR (Student’s *t* test). **g** qRT-PCR analysis of miR-203 expression in RASFs transfected with pcNDA, circ_0000396, si-NC, or si-circ_0000396 was conducted (Student’s *t* test). ^*^p < 0.05, ^**^p < 0.01, ^***^p < 0.001, ^****^p < 0.0001

It has been reported that circRNAs can function as sponges of microRNAs (miRNAs) to regulate gene expression [17]. Thus, to study the mechanism underlying the action of circ_0000396 on RASF dysfunction, the direct miRNA targets of circ_0000396 were predicted by searching circinteractome database, and the binding sites between miR-203 and circ_0000396 were presented (Fig. 3a). Afterwards, a dual luciferase reporter assay was performed and a decline of luciferase activity in RASFs co-transfected with circ_0000396 WT plasmids and miR-203 was observed, but there was no obvious impact on circ_0000396 MUT plasmids compared with the control group after up-regulation of miR-203 expression in cells (Fig. 3b). Meanwhile, we also found that the inhibition of miR-203 enhanced the luciferase activity of the circ_0000396 WT reporter vector but not mutant reporter vector in RASFs (Fig. 3c). Additionally, the data of pull-down analysis exhibited a significant enrichment of circ_0000396 in Bio-miR-203 group relative to Bio-miR-203 MUT or Bio-miR-NC group (Fig. 3d). These data confirmed that circ_0000396 directly bound to miR-203 in RASFs. After that, the expression of miR-203 was analyzed; qRT-PCR analysis showed miR-203 was up-regulated in RA synovial tissues and RASFs relative to the normal controls (Fig. 3e, f), and miR-203 was overexpressed in RASFs when circ_0000396 was down-regulated, but was decreased when circ_0000396 was up-regulated (Fig. 3g). Therefore, circ_0000396 negatively regulated miR-203 expression in RASFs.

### Circ_0000396 affects the growth and inflammation of RASFs by binding to miR-203

**Fig. 4:**
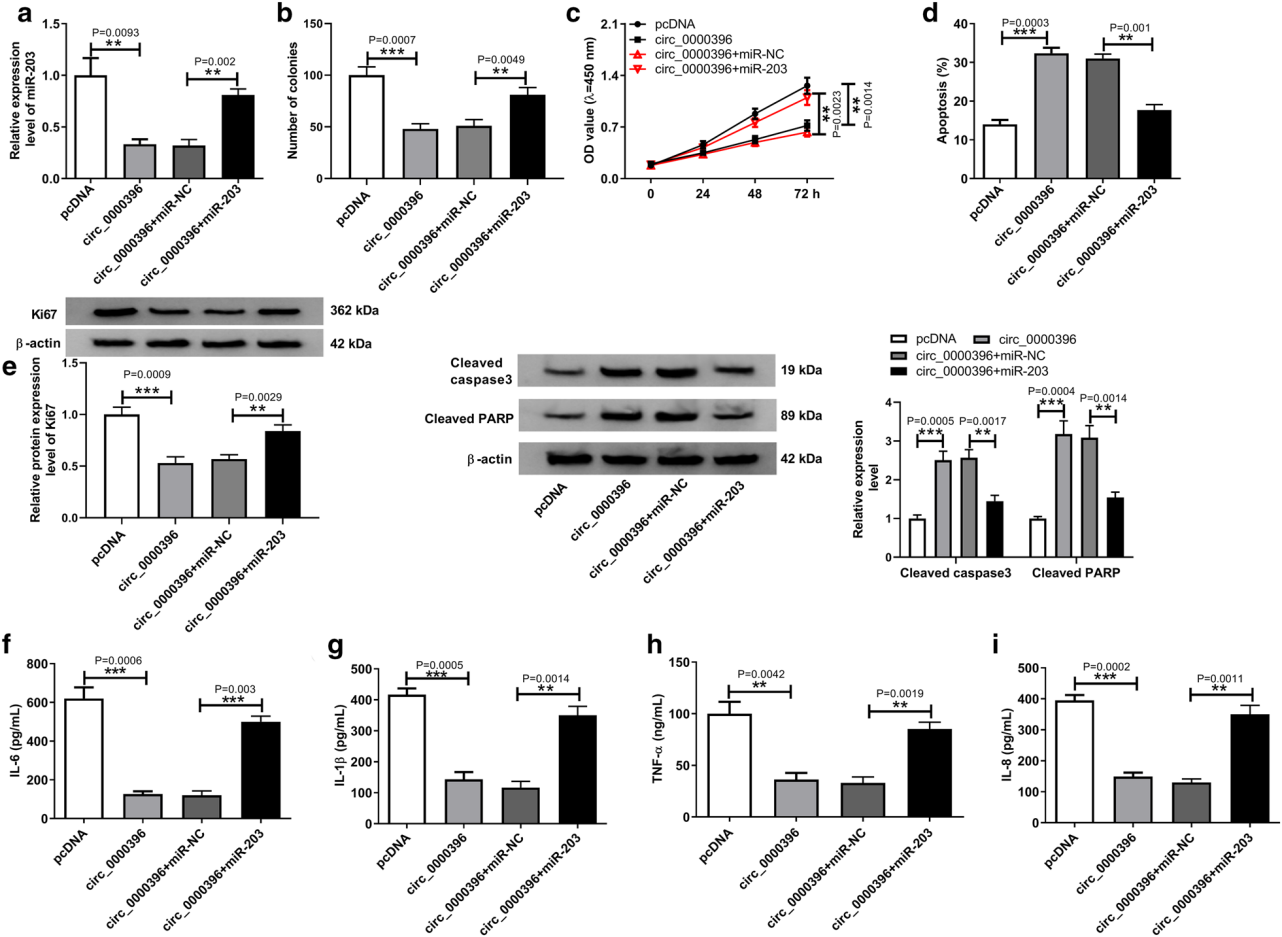
Circ_0000396 affects the growth and inflammation of RASFs by binding to miR-203. RASFs were transfected with pcDNA, circ_0000396, circ_0000396 + miR-NC, circ_0000396 + miR-203. After transfection, **a** the expression of miR-203 was measured by qRT-PCR; **b**, **c** RASF proliferation was analyzed by colony formation assay and CCK-8 assay, respectively; **d** the apoptosis of RASFs was measured by flow cytometry; **e** the expression of Ki67, cleaved caspase3 and cleaved PARP expression in RASFs was detected using western blot; **f**–**i** The level of inflammatory cytokines IL-6, IL-1β, IL-8 and TNF-α in RASFs was detected by ELISA assay. ^*^p < 0.05, ^**^p < 0.01, ^***^p < 0.001, ^****^p < 0.0001 (ANOVA)

We further explored whether miR-203 involved in the protective functions of circ_0000396 on RASFs malignant changes and inflammation. First, RASFs were transfected with pcDNA, circ_0000396, circ_0000396 + miR-NC, circ_0000396 + miR-203. After transfection, we found that the expression of miR-203 was inhibited by circ_0000396 overexpression, which was rescued by miR-203 mimic (Fig. 4a), indicating the successful transfection. Afterwards, rescue assay was conducted, and results displayed miR-203 overexpression reversed circ_0000396 restoration-mediated inhibition of proliferation (Fig. 4b, c, e), and promotion of apoptosis (Fig. 4d, e) in RASFs. In addition, ELISA analysis showed the massive release of inflammatory cytokines IL-6, IL-1β, IL-8, and TNF-α induced by circ_0000396 in RASFs was also attenuated by miR-203 up-regulation (Fig. 4f–i). In all, circ_0000396 regulated RA progression by interacting with miR-203.

### Circ_0000396 can directly regulate miR-203 targeted gene HBP1 in RASFs

**Fig. 5:**
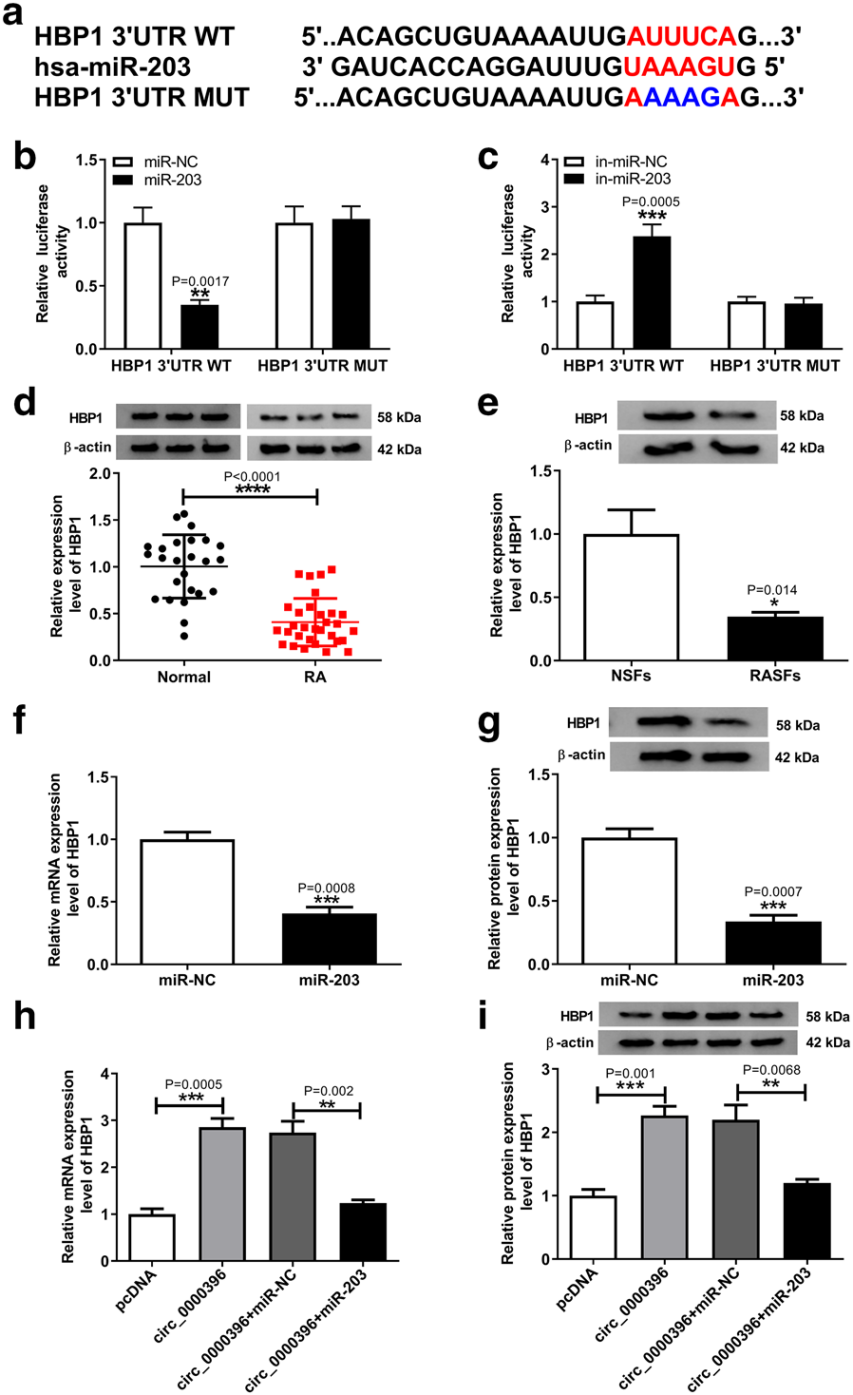
Circ_0000396 can directly regulate miR-203 targeted gene HBP1 in RASFs. **a** The putative binding sits between HBP1 3΄UTR and miR-203 were listed. **b**, **c** The interaction between circ_0000396 and miR-203 was confirmed by the dual-luciferase reporter assay in RASFs (Mann–Whitney U-test). **d**, **e** The protein expression of HBP1 in RA synovial tissues and healthy control tissues, as well as in RASFs and NSFs was detected by western blot (Student’s *t* test). **f**, **g** qRT-PCR or western blot analysis of HBP1 expression in RASFs transfected with miR-NC, or miR-203 was conducted (Student’s *t* test). **h**, **i** Levels of HBP1 in RASFs transfected pcDNA, circ_0000396, circ_0000396 + miR-NC, circ_0000396 + miR-203 were detected by qRT-PCR or western blot (ANOVA). ^*^p < 0.05, ^**^p < 0.01, ^***^p < 0.001, ^****^p < 0.0001

Through searching the online program DIANA TOOLS, HBP1 was found to have the potential binding sites in miR-203 (Fig. 5a). Immediately, the reduction of luciferase activity in RASFs co-transfected with HBP1 3΄UTR WT vectors and miR-203 (Fig. 5b), and the increase of luciferase activity in RASFs co-transfected with HBP1 3΄UTR WT vectors and in-miR-203 (Fig. 5c) confirmed the direct interaction between HBP1 and miR-203. After that, qRT-PCR analysis showed HBP1 expression was both decreased in RA synovial tissues and RASFs (Fig. 5d, e), and miR-203 inhibited the expression of HBP1 in RASFs (Fig. 5f, g). Moreover, circ_0000396 was also discovered to suppress HBP1 expression at mRNA and protein levels, but this inhibition could be abated by overexpressed miR-203 in RASFs (Fig. 5h, i). In summary, HBP1 was a target of miR-203, and circ_0000396 could directly regulate HBP1 expression by binding to miR-203 in RASFs.

### HBP1 silence reverses the function of miR-203 on RASF growth and inflammation

**Fig. 6:**
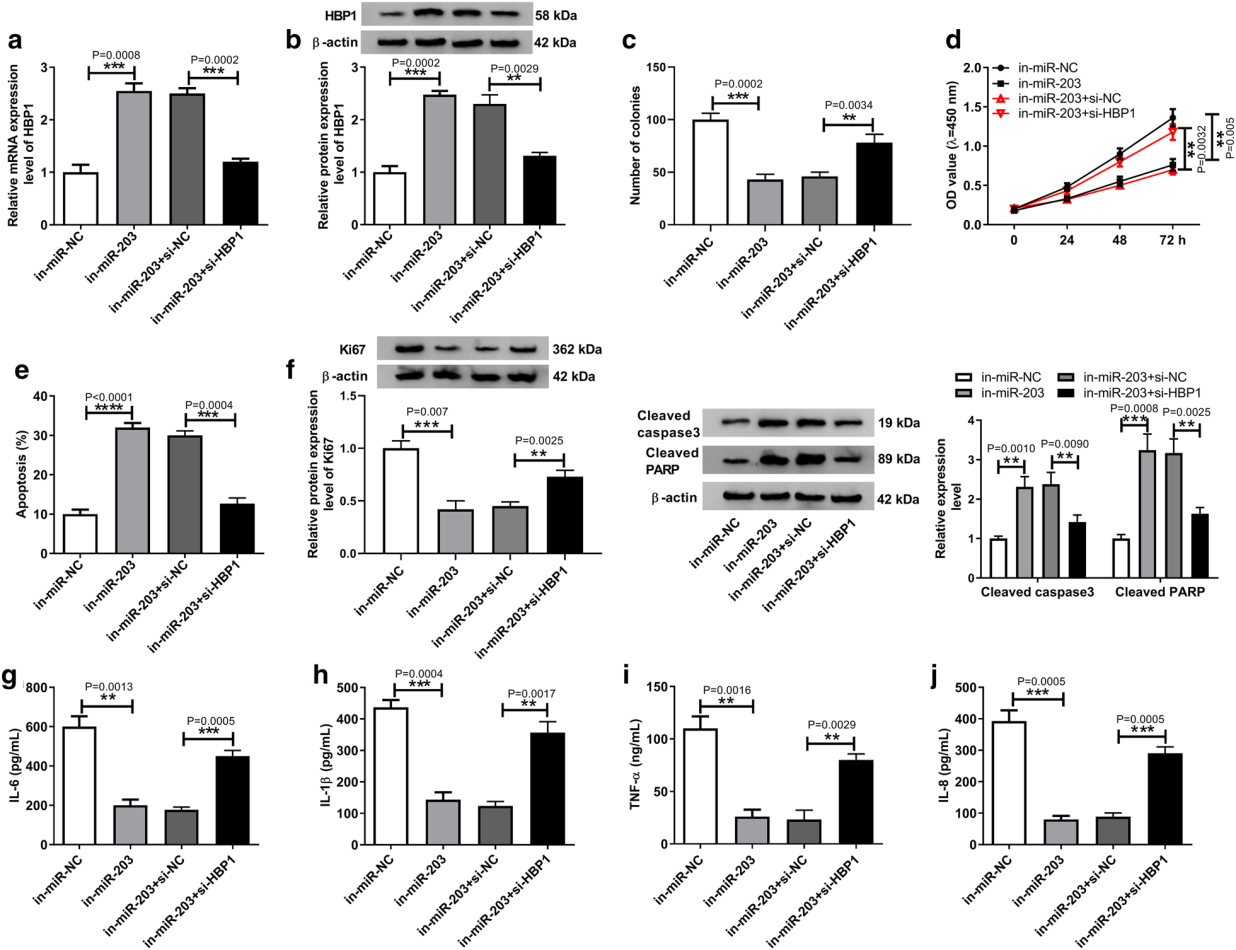
HBP1 silence reverses the function of miR-203 on RASF growth and inflammation. RASFs were transfected with in-miR-NC, in-miR-203, in-miR-203 + si-NC, or in-miR-203 + si-HBP1. **a**, **b** Transfection efficiency was analyzed by using qRT-PCR or western blot. **c**, **d** The proliferation of RASFs was determined by colony formation assay and CCK-8 assay, respectively. **e** Flow cytometry was applied to detect the apoptosis of RASFs. **f** Western bolt analysis of Ki67, cleaved caspase3 and cleaved PARP expression in RASFs was carried out. **g**–**j** The level of inflammatory cytokines IL-6, IL-1β, IL-8 and TNF-α in RASFs was detected by ELISA assay. ^*^p < 0.05, ^**^p < 0.01, ^***^p < 0.001, ^****^p < 0.0001 (ANOVA)

The effects of miR-203 on RASFs were investigated. RASFs were transfected with in-miR-NC, in-miR-203, in-miR-203 + si-NC, or in-miR-203 + si-HBP1. Then, results showed that HBP1 expression was promoted by miR-203 inhibition, which was rescued by HBP1 knockdown in RASFs at mRNA and protein levels (Fig. 6a, b), suggesting the successful transfection. Subsequently, we found miR-203 inhibition mediated proliferation arrest (Fig. 6c, d, f), apoptosis elevation (Fig. 6e, f), and release suppression of inflammatory cytokines (Fig. 6g–j) in RASFs; however, these functions could be reversed by HBP1 silencing (Fig. 6b–j). Altogether, miR-203 inhibition suppressed the growth and inflammation of RASFs by regulating HBP1.

### Circ_0000396 affects the growth and inflammation of RASFs through modulating HBP1

**Fig. 7:**
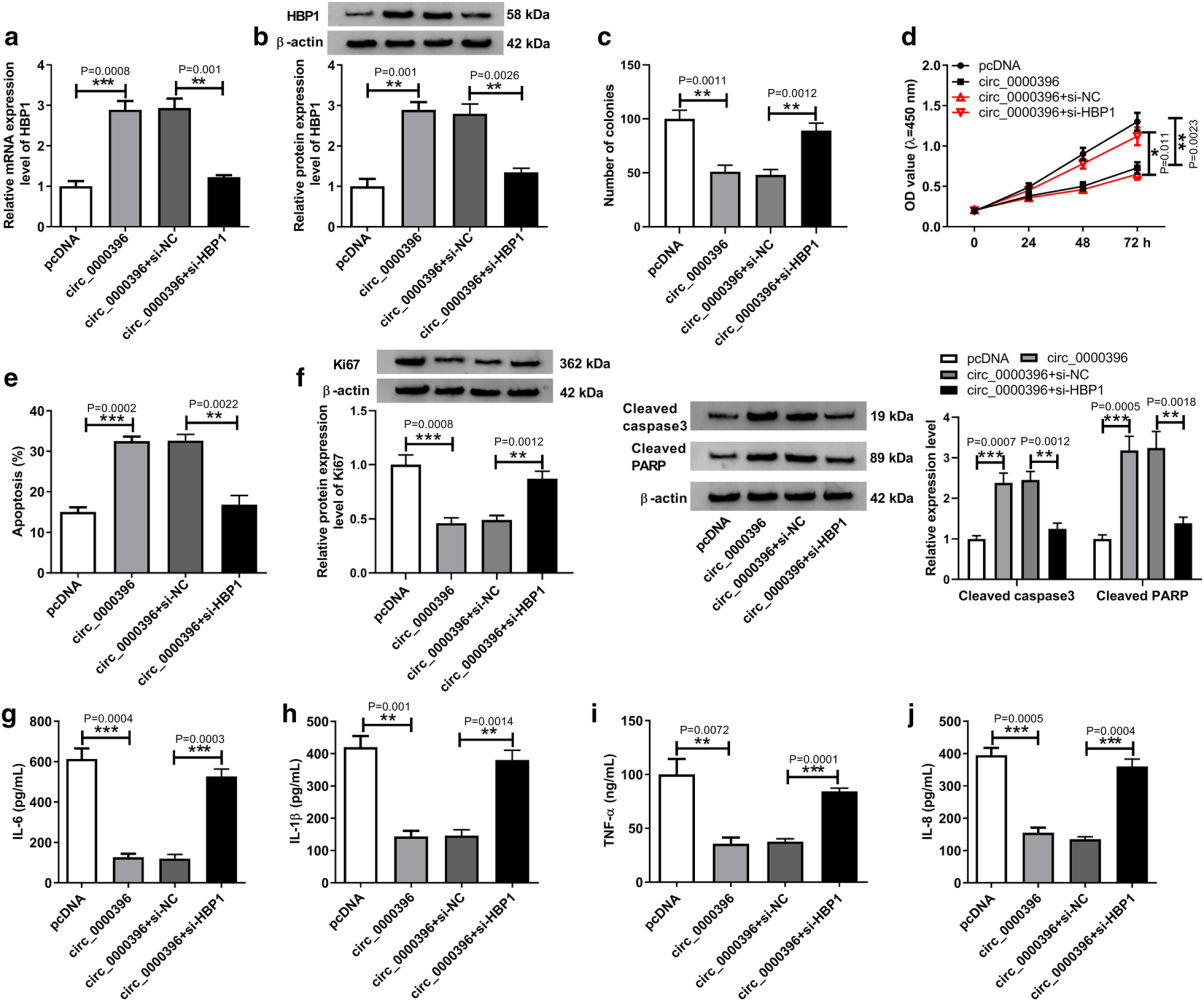
Circ_0000396 affects the growth and inflammation of RASFs through modulating HBP1. RASFs were transfected with pcDNA, circ_0000396, circ_0000396 + si-NC, or circ_0000396 + si-HBP1. **a**, **b** Transfection efficiency was analyzed by using qRT-PCR or western blot. **c**, **d** The proliferation of RASFs was determined using colony formation assay and CCK-8 assay. **e** The apoptosis rate of RASFs was detected using flow cytometry. **f** The expression of Ki67, cleaved caspase3 and cleaved PARP expression in RASFs was detected using western blot. **g**–**j** Levels of inflammatory cytokines IL-6, IL-1β, IL-8 and TNF-α in RASFs was detected by ELISA assay. ^*^p < 0.05, ^**^p < 0.01, ^***^p < 0.001, ^****^p < 0.0001 (ANOVA)

Given that circ_0000396 could regulate HBP1 expression via miR-203, whether circ_0000396 performed protective effects on RASFs by regulating HBP1 was investigated. RASFs were transfected with pcDNA, circ_0000396, circ_0000396 + si-NC, or circ_0000396 + si-HBP1, then we found si-HBP1 attenuated circ_0000396-mediated up-regulation of HBP1 expression in RASFs (Fig. 7a, b). Subsequently, rescue assay was conducted, results exhibited that knockdown of HBP1 overturned circ_0000396-induced inhibition on malignant phenotypes and inflammation, evidenced by the increase of RASF proliferation (Fig. 7c, d, f) and decrease of apoptotic RASFs (Fig. 7e, f), as well as the release of IL-6, IL-1β, IL-8, and TNF-α in RASFs (Fig. 7g–j). Thus, we conformed that circ_0000396 regulated RA progression by modulating HBP1.

## Discussion

Growing studies have indicated that circRNAs are important regulators in complex human pathologies, and circRNAs are found to play a vital role in the immune system, and are greatly connected with the onset and progression of autoimmune diseases, including RA [18]. In this study, we also found circ_0000396 was decreased in RA synovial tissues and RASFs, which was consistent with previous study [16]. Then, functional experiments showed circ_0000396 overexpression inhibited cell proliferation and induced cell apoptosis in RASFs, while circ_0000396 decrease showed opposite effects. Besides, we also found the secretion of IL-6, IL-1β, IL-8, and TNF-α inflammatory cytokines was suppressed by overexpressed circ_0000396, thus suppressing inflammation in RASFs.

RASF stimulation is a pivotal factor in the transform of affected synovium to healthy synovium, thus inducing the expansion of arthritis and distant joints destruction [19, 20]. It has been shown RA is closely associated with the abnormal hyperplasia of RASFs [21]. RASFs can produce plentiful cytokines and matrix metalloproteinases (MMPs) that, in turn, stimulate the migration and invasion of themselves [19]. In addition, proliferation elevation combined with insufficient apoptosis may also induce the spread of RASFs, giving rise to the bone destruction [22]. Importantly, RASF proliferation also results in the immunoreaction, and ultimately contributes to the joint damage [23]. RA patients always highly express inflammatory cytokines, such as IL-6, IL-1β, IL-8 and TNF-α, which activate STAT3 either directly or indirectly, and in turn boosts the expression of these inflammatory cytokines in an autocrine manner in RASFs. Besides, STAT3 activation also stimulates the expression of receptor activator of nuclear factor kappa B ligand (RANKL), which is necessary for osteoclastogenesis; all these changes further promoted chronic inflammation and joint destruction [24, 25]. Thus, circ_0000396 may be a promising therapeutic target to prevent RA progression.

It is well believed that circRNAs act as miRNA sponges to regulate gene expression [17]. MiRNAs are small non-protein coding RNAs, and have been classified to play a multifunctional role in the pathogenesis of RA. For example, miR-20a inhibited proliferation and induced apoptosis of RASFs by down-regulating the expression of STAT3 [26]. MiR-338-5p contributed to the promotion of proliferation, invasiveness and the release of pro-inflammatory cytokines in RASFs by mediating the inhibition of SPRY1 [27]. MiR-650 interacted with AKT2 to suppress the biological behaviors of RASFs to impede the progression of RA [28]. In our study, we confirmed circ_0000396 directly targeted miR-203. MiR-203 was increased in RA synovial tissues and RASFs, and inhibition of miR-203 suppressed cell growth and inflammation in RASFs. Besides, miR-203 overexpression reversed the protective effects of circ_0000396 on RASFs dysfunction.

We also confirmed miR-203 directly bound to HBP1. HBP1 is a ubiquitous transcription factor, and plays significant roles in senescence, apoptosis, differentiation termination, and tumor suppression in various cell types [29]. HBP1 can accelerate cell death through up-regulating the expression of p21, an imperative mediator of p53-dependent apoptosis, to reinforce the stability of p53 by repressing p53 ubiquitination induced by Mdm2 [30]. Chen et al. [31] revealed that HBP1 decreased the expression of macrophage migration inhibitory factor (MIF), an important pro-inflammatory chemokine, to hinder the release of inflammatory cytokines TNF-α and IL-6. Consistently in the current study, HBP1 was decreased in RA synovial tissues and RASFs, and HBP1 knockdown attenuated miR-203 inhibition-mediated dysfunction and inflammation in RASFs. In addition, we also observed circ_0000396 indirectly regulated HBP1 expression through binding to miR-203, and knockdown of HBP1 reversed the protective effects of circ_0000396 on RASF dysfunction. In all, these results suggest a promising therapeutic target for the molecular therapy of RA patients. However, the function of circ_0000396 in healthy cell lines should be detected before the application of circ_0000396 to clinical use, in order to ensure the safety and efficiency. Additionally, the delivery methods of RNA remain the challenges for clinical application.

## Conclusion

**Fig. 8:**
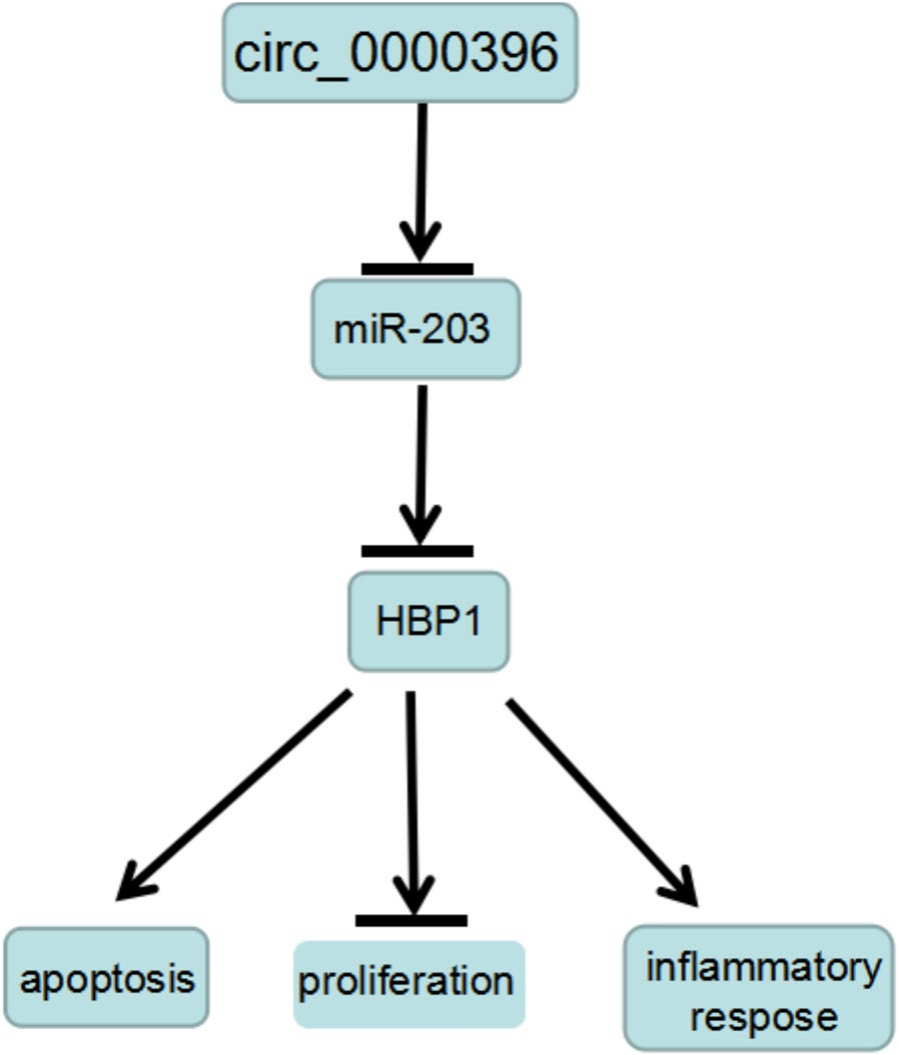
Schematic model showing the role of circ_0000396 in the growth and inflammation of RASFs. Circ_0000396 could suppress RASF growth and inflammation by up-regulating HBP1 through absorbing miR-203

In conclusion, our findings demonstrated circ_0000396 could suppress RASF growth and inflammation by up-regulating HBP1 through miR-203 (Fig. 8), which suggested a novel idea into understanding the pathogenesis of RA process. Nevertheless, there are still some limitations. The data presented are based on a limited number of cells in vitro, in vivo assay and a larger cohort of the disease analysis are necessary for the verification of these conclusions in the future. Moreover, the potent, long-lasting, and safe immune responses of circ_0000396 in animal models should be investigated before clinical application.

## Supplementary Information

**Additional file 1: Fig. S1.** Identification of RASFs and NSFs by flow cytometry. The purity of the third generation of RASFs (A) and NSFs (B) of the primary culture reached 98.0% and 99.0%, respectively.

## Data Availability

The datasets used and/or analysed during the current study are available from the corresponding author on reasonable request.
