# Supplementary figures and images for: Circ_0000396 inhibits rheumatoid arthritis synovial fibroblast growth and inflammatory response via miR-203/HBP1 axis
Source: J Biol Res (Thessalon). 2021 Jan 6;28:1. doi: 10.1186/s40709-020-00131-4 (PMC7788801; doi:10.1186/s40709-020-00131-4)

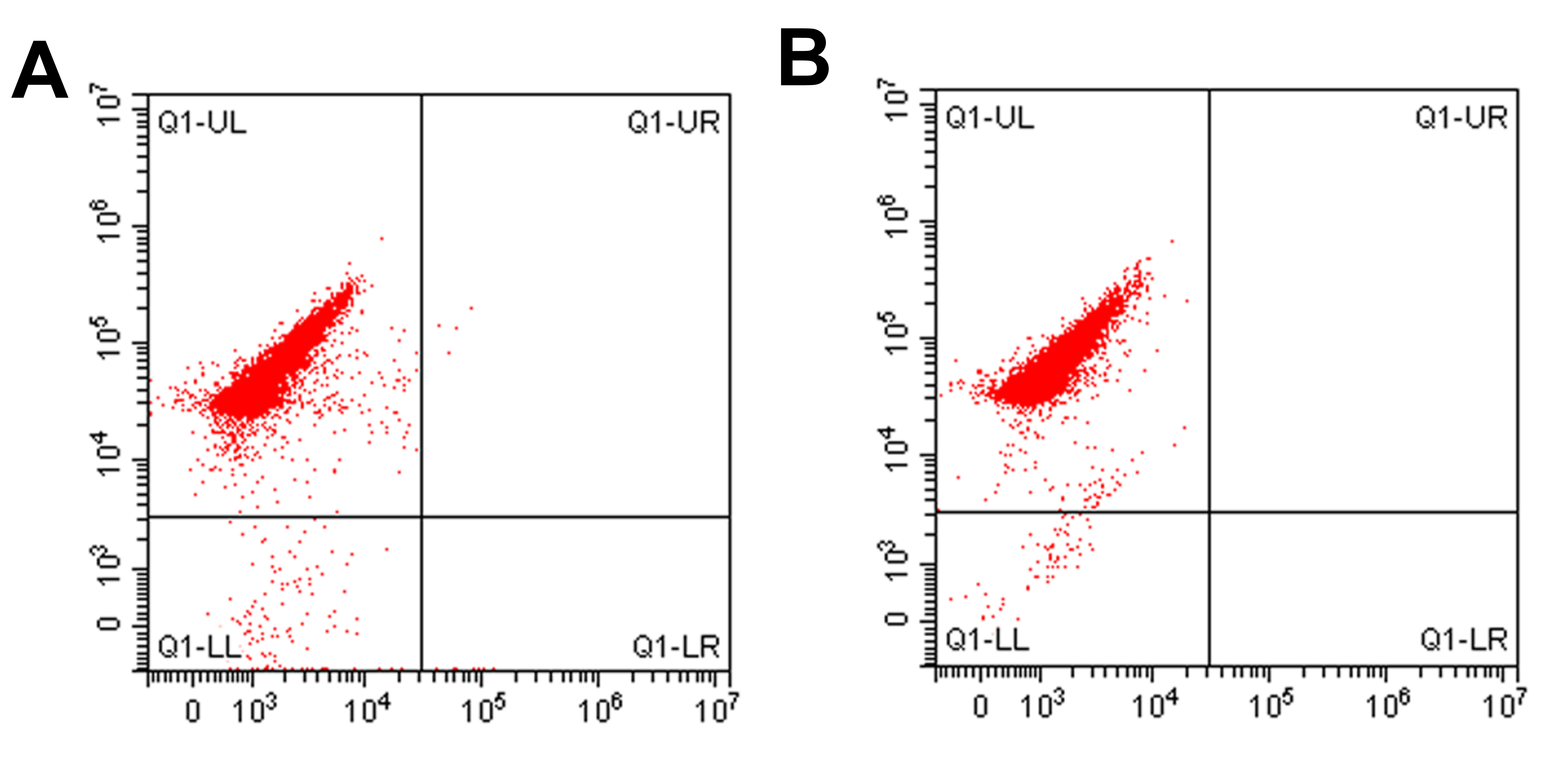

Supplement: Supplementary file 1 — Additional file 1: Fig. S1. Identification of RASFs and NSFs by flow cytometry. The purity of the third generation of RASFs (A) and NSFs (B) of the primary culture reached 98.0% and 99.0%, respectively. [file 40709_2020_131_MOESM1_ESM.tif]
